# Effects of *Ruscus aculeatus* Rhizome Extract on Human Cerebral Microvascular Endothelial Cells Under Normoxic and Hypoxic Conditions

**DOI:** 10.3390/molecules31183348

**Published:** 2026-09-21

**Authors:** Magdalena Wójciak, Magdalena Żuk, Marcin Feldo, Zenon Czuba, Anna Kurek-Górecka, Ireneusz Sowa

**Affiliations:** 1 Department of Analytical Chemistry, Medical University of Lublin, Chodźki 4a, 20-093 Lublin, Poland; magdalena.zu25@gmail.com; 2 Chair and Department of Vascular Surgery and Angiology, Medical University of Lublin, 8 Solidarności St., 20-841 Lublin, Poland; martinf@interia.pl; 3 Department of Microbiology and Immunology, Medical University of Silesia in Katowice, Jordana 19, 41-808 Zabrze, Poland; zczuba@sum.edu.pl (Z.C.); akurekgorecka@sum.edu.pl (A.K.-G.)

**Keywords:** *Ruscus aculeatus*, butcher’s broom, endothelial cells, hypoxia, steroidal saponins, cytokines, TGF-β, vascular homeostasis

## Abstract

*Ruscus aculeatus* L. (butcher’s broom) is traditionally used in the management of chronic venous disorders; however, the molecular mechanisms underlying its vascular activity remain insufficiently characterized. This study investigated the effects of an 80% ethanolic rhizome extract on human cerebral microvascular endothelial hCMEC/D3 cells under normoxic and hypoxic conditions. The extract was phytochemically characterized using ultra-high-performance liquid chromatography–diode array detection–high-resolution mass spectrometry (UHPLC–DAD–HRMS), while its biological effects were evaluated by assessing cell viability and the secretion of selected cytokines, including transforming growth factor beta (TGF-β) isoforms, using Bio-Plex multiplex assays. Phytochemical analysis confirmed steroidal saponins as the predominant constituents and also revealed minor phenolic compounds, including hydroxycinnamic acid amides. The extract (10 and 50 μg/mL) was well tolerated by hCMEC/D3 cells and did not significantly reduce cell viability under either normoxic or hypoxic conditions. Its effects on cytokine secretion depended on oxygen availability and the mediator examined. Under hypoxia, the extract partially shifted interleukin-6 (IL-6) and interleukin-8 (IL-8) secretion toward normoxic levels and attenuated interferon alpha (IFN-α)-induced tumor necrosis factor alpha (TNF-α) production, whereas a significant increase in granulocyte-macrophage colony-stimulating factor (GM-CSF) relative to the untreated control was observed only with the combination of 50 μg/mL extract and IFN-α. Hypoxia also differentially affected TGF-β isoforms, increasing TGF-β1 and decreasing TGF-β2, while treatment with the extract shifted both responses toward the profile observed under normoxia; TGF-β3 remained largely unchanged. Overall, these findings indicate that *R. aculeatus* rhizome extract does not exert a uniform anti-inflammatory effect but rather modulates endothelial secretory responses in an oxygen- and mediator-dependent manner. These observations provide new insights into the endothelial effects of *R. aculeatus* that may contribute to its vascular activity.

## 1. Introduction

*Ruscus aculeatus* L. (butcher’s broom) is a perennial evergreen shrub native to the Mediterranean region and parts of Western and Central Europe. For centuries, it has been used in traditional medicine for the management of chronic venous insufficiency, hemorrhoids, and disorders associated with impaired peripheral circulation [1,2,3]. Today, extracts of *R. aculeatus* remain an important component of several registered medicinal products, including combination formulations such as Cyclo 3 Fort, which are widely prescribed for the treatment of chronic venous disease and related vascular disorders [4]. The therapeutic value of *R. aculeatus* is primarily attributed to its well-documented venotonic and vasoprotective properties. Extensive experimental and clinical research has demonstrated that *R. aculeatus* extracts improve venous tone, reduce capillary permeability, alleviate edema, and enhance lymphatic function [5]. Consequently, most published studies have focused on vascular and lymphatic physiology using ex vivo vascular preparations, animal models of venous dysfunction, and clinical investigations in patients with chronic venous insufficiency [6]. Despite its long history of medicinal use and the evidence supporting its clinical efficacy, the molecular mechanisms underlying the biological activity of *Ruscus aculeatus* remain only partially understood [7,8,9]. In particular, little is known about its direct effects on endothelial cell function under pathological conditions associated with hypoxia and inflammation [8]. The vascular endothelium plays a central role in maintaining cardiovascular homeostasis by regulating vascular tone, permeability, hemostasis, leukocyte trafficking, angiogenesis, and inflammatory responses [10]. Consequently, endothelial dysfunction, characterized by impaired barrier integrity and dysregulated production of inflammatory mediators, is recognized as an early event in the development of numerous cardiovascular diseases, including chronic venous disease [10]. Hypoxia, a common feature of many cardiovascular disorders, is one of the major triggers of endothelial activation and dysfunction. Reduced oxygen availability stabilizes hypoxia-inducible factor-1α (HIF-1α), leading to altered expression of numerous genes involved in inflammation, angiogenesis, and cellular adaptation [11,12].

In this context, the present study aimed to investigate the effects of a *Ruscus aculeatus* rhizome extract on endothelial responses under normoxic and hypoxic conditions. For this purpose, the human cerebral microvascular endothelial cell line (hCMEC/D3), a well-characterized in vitro model of human microvascular endothelial cells, was employed. Microvascular endothelial cells actively contribute to vascular homeostasis, inflammatory signaling, cytokine production, and the regulation of endothelial permeability. Therefore, this model provides a biologically relevant platform for investigating the effects of natural products on endothelial responses, particularly under hypoxic conditions associated with endothelial dysfunction. To ensure the relevance of the study, a dry extract prepared from *R. aculeatus* rhizomes using 80% ethanol, corresponding to extracts employed in commercially available medicinal products, was selected. Prior to the biological evaluation, the extract was characterized with respect to its major bioactive constituents, including steroidal saponins and polyphenolic compounds. The findings of this study are expected to provide new insights into the endothelial activity of *R. aculeatus.*

## 2. Results and Discussion

### 2.1. Phytochemical Characterisation of the Extract

**Figure 1:**
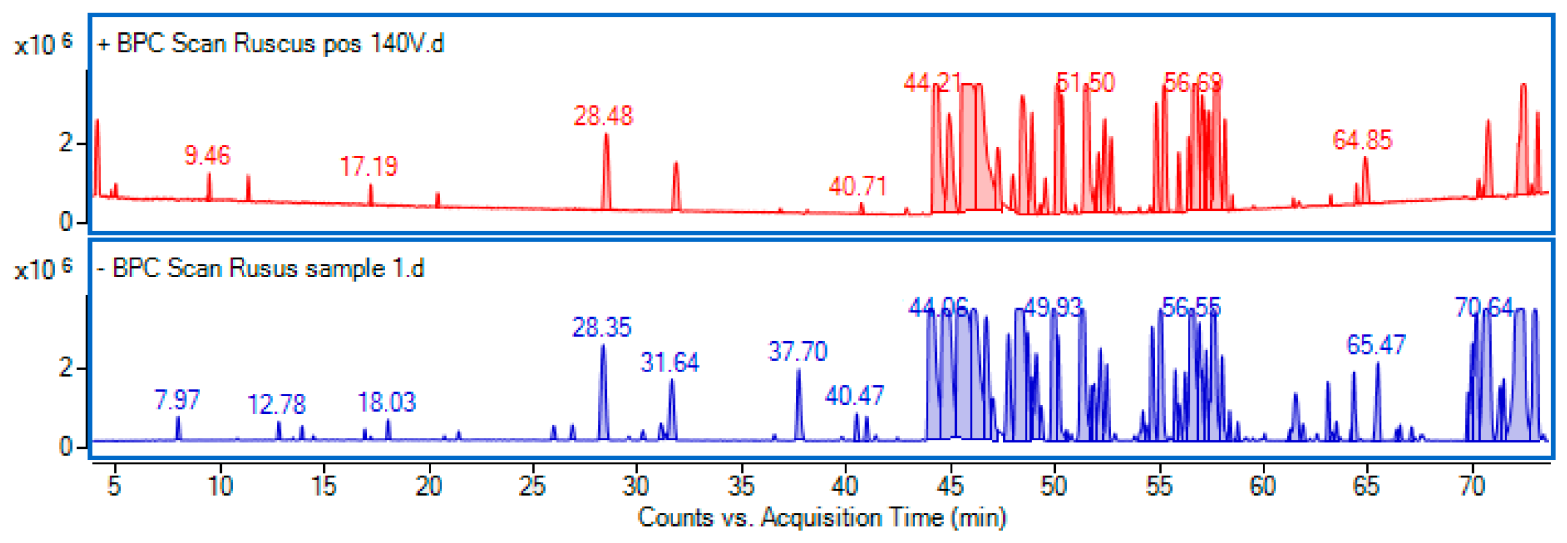
UHPLC–HRMS chromatographic profiles of the *Ruscus aculeatus* rhizome extract recorded in positive-ion mode (red line) and negative-ion mode (blue line). Compound names corresponding to retention times are provided in Table 1 and Table 2.

Phytochemical characterization of the *R. aculeatus* extract was performed using ultra-high-performance liquid chromatography–diode array detection–high-resolution mass spectrometry (UHPLC–DAD–HRMS) operated in both positive- and negative-ionization modes (Figure 1).

**Table 1:** Chromatographic and high-resolution mass spectrometric characteristics of phenolic compounds detected in the *R. aculeatus* extract.

Rt (min)	Formula	ESI (+), m/z		ESI (−), m/z		Tentative Identification
		[M+Na]^+^/[M+H]^+^	[(M−H_2_O)+H]^+^	[M−H]^−^	[M−H−H_2_O]^−^	
7.97	C_8_H_10_O_3_	-	-	153.05588 (123)	-	Hydroxytyrosol *
28.48	C_17_H_17_NO_4_	322.1045/300.1234	282.1143	298.10899	280.0981	*N*-*p*-coumaroyloctopamine/dopamine
31.83	C_18_H_19_NO_5_	352.1147/330.1337	312.1235	328.11972	310.1085	*N*-feruloyloctopamine/dopamine
44.96	C_17_H_17_NO_3_	306.1088/284.1286	-	282.1143	-	*N*-*p*-coumaroyltyramine
47.06	C_18_H_19_NO_4_	336.1197/314.1392	-	312.1251	-	*N*-feruloyltyramine

*—confirmed by standard.

**Table 2:** Chromatographic and high-resolution mass spectrometric characteristics of saponins and related compounds detected in the *R. aculeatus* extract.

No	Rt (min)	Formula	ESI (+), m/z		ESI (−), m/z	
			[M+Na]^+^/[M+H]^+^ *	[(M−H_2_O)+H]^+^	[M−H]^−^	[M+HCOO]^−^
1	44.21	C_50_H_80_O_23_	1071.4983	1031.5058	1047.5018	1093.5067
2	44.91	C_47_H_66_O_21_	-	949.406	965.40566	-
3	45.02	C_44_H_70_O_18_	909.4454	869.4529	885.4459	931.4524
4	46.36	C_44_H_72_O_18_	911.4611	871.4686	887.4646	933.4695
5	48.44	C_49_H_68_O_22_	-	991.417	1007.4168	-
6	48.89	C_52_H_82_O_24_	1113.5088	1073.5163	1089.5123	1135.5173
7	50.14	C_52_H_82_O_24_	1113.5088	1073.5163	1089.5123	1135.5173
8	50.32	C_46_H_72_O_19_	951.4560	911.4635	927.4595	973.4644
9	51.50	C_46_H_72_O_19_	951.4560	911.4635	927.4595	973.4644
10	52.37	C_50_H_80_O_21_	1039.5059	999.5151	1015.5146	1061.5194
11	55.22	C_54_H_84_O_25_	-	1115.5259	1131.52753	-
12	56.69	C_50_H_80_O_20_	1023.5135	983.5210	999.5170	1045.5219
13	57.05	C_50_H_82_O_20_	1025.5263	985.5486	1001.5326	1047.5392
14	57.17	C_50_H_80_O_20_	1023.5123	983.5301	999.5168	1045.5215
15	57.39	C_51_H_80_O_22_	1067.5019	1027.5116	1043.5089	1089.5162
16	57.72	C_52_H_82_O_21_	1065.5241	1025.5316	1041.5276	1087.5325
17	58.14	C_51_H_80_O_22_	1067.5021	1027.5124	1043.5097	1089.5173
18	70.75	C_34_H_58_O_20_	787.35681 *	-	785.34441	-
19	72.43	C_38_H_58_O_12_	707.4001 *	–-	705.3856	751.3905
20	73.11	C_38_H_60_O_12_	709.4158 *	-	707.4012	753.4061

* Values marked with an asterisk correspond to [M+H]^+^ ions; unmarked values correspond to [M+Na]^+^ ions.

The chromatographic profile was dominated by steroidal saponins, whereas the early-eluting, highly polar fraction contained relatively few detectable constituents. Only a limited number of peaks exhibited UV–Vis absorption in the wavelength range characteristic of phenolic compounds, suggesting that this fraction consisted predominantly of low-molecular-weight polar metabolites lacking extended conjugated chromophoric systems. Some minor constituents were tentatively identified. Hydroxytyrosol was tentatively annotated based on the characteristic deprotonated molecular ion at m/z 153, the diagnostic fragment ion at m/z 123, and its UV–Vis spectrum displaying two absorption maxima at approximately 225 and 280 nm. In addition, four hydroxycinnamic acid amides (phenolamides), representing conjugates of hydroxycinnamic acids with aromatic amines, were tentatively identified based on accurate mass measurements, characteristic fragmentation patterns, and comparison with literature data reported by Li et al. [13]. The compound eluting at Rt = 28.0 min was tentatively assigned as *N*-*p*-coumaroyloctopamine or *N*-*p*-coumaroyldopamine, with a content of 0.76 mg/g of dried extract. The presence of dehydrated ions in both positive- and negative-ion modes suggested that the octopamine derivative was more likely. The peak detected at Rt = 41.9 min was tentatively assigned as *N*-*p*-coumaroyltyramine and was present at 0.51 mg/g of dried extract. Both compounds produced the characteristic product ion at m/z 147 in positive-ion mode, indicative of the *p*-coumaroyl moiety. Likewise, the compounds eluting at Rt = 31.83 min and Rt = 47.06 min were tentatively identified as *N*-feruloyloctopamine (or *N*-feruloyldopamine) and *N*-feruloyltyramine, respectively, with corresponding contents of 0.62 and 0.25 mg/g of dried extract. The dehydrated molecular ions observed for the compound at Rt = 31.83 min likewise favored the octopamine derivative, while the product ion at m/z 177 was characteristic of the feruloyl substructure.

It should be noted that information on the phenolic constituents of *R. aculeatus* rhizomes remains very limited. In the study by Hadžifejzović et al., the authors reported the presence of *p*-coumaric acid and hydroxycinnamic acid amides [14]; however, these findings were cited only with reference to an unpublished doctoral thesis, and no analytical data were provided. In the present study, representative MS and UV–Vis spectra of the major hydroxycinnamic acid amides are included in the Supplementary Materials (Figure S1), providing additional analytical characterization of compounds previously reported in *R. aculeatus* rhizomes.

The major constituents of the extract were steroidal saponins, which are recognized as the characteristic specialized metabolites of *Ruscus* species. Their tentative characterization was based on accurate mass measurements, retention behavior, and characteristic ionization patterns observed in both positive- and negative-ion modes. Most compounds exhibited ionization behavior consistent with furostanol-type steroidal saponins. In positive ESI mode, they were detected predominantly as sodium adducts ([M+Na]^+^) together with dehydrated protonated molecules ([(M−H_2_O)+H]^+^), the latter reflecting the characteristic dehydration of the C-22 hydroxyl group of 22-hydroxyfurostanol derivatives. In negative ESI mode, these compounds generally produced deprotonated molecules ([M−H]^−^), frequently accompanied by formate adducts ([M+HCOO]^−^). Notably, several constituents generated only dehydrated protonated ions in positive mode together with deprotonated molecules in negative mode, while maintaining chromatographic behavior consistent with steroidal saponins. In contrast, three late-eluting compounds (above Rt 70 min) exhibited abundant sodium adducts without dehydration in positive-ion mode, together with [M−H]^−^ and [M+HCOO]^−^ ions in negative-ion mode, a pattern characteristic of spirostanol-type saponins.

The chromatographic and high-resolution mass spectrometric characteristics of all detected metabolites are summarized in Table 1.

Saponins with similar spectral data, molecular masses, and molecular formulas have been previously reported by Mari et al. [15] and Festa et al. [16]. Compound no. **3**, tentatively identified as Ruscoponticoside E, produced the most intense signal among the detected saponins in both the DAD and MS chromatographic profiles (Figure S2).

### 2.2. Biological Assay

Hypoxia is a key pathological factor contributing to endothelial dysfunction, inflammation, impaired microcirculation, and vascular remodeling. Therefore, experiments were conducted under both normoxic and hypoxic conditions to determine whether *Ruscus aculeatus* extract modulates endothelial responses under hypoxic stress. The hCMEC/D3 cell line was selected as a well-characterized and reproducible human microvascular endothelial model suitable for investigating direct endothelial responses to hypoxic and inflammatory stimuli. The use of an established endothelial cell line reduces donor-dependent variability associated with primary endothelial cultures and facilitates standardized comparisons across experimental conditions. Although hCMEC/D3 cells originate from cerebral microvessels and therefore do not directly reproduce peripheral venous endothelium, they remain a valuable model for studying mechanisms associated with endothelial activation and adaptation to hypoxic stress.

#### 2.2.1. Effect of *Ruscus aculeatus* Extract on hCMEC/D3 Cell Viability

**Figure 2:**
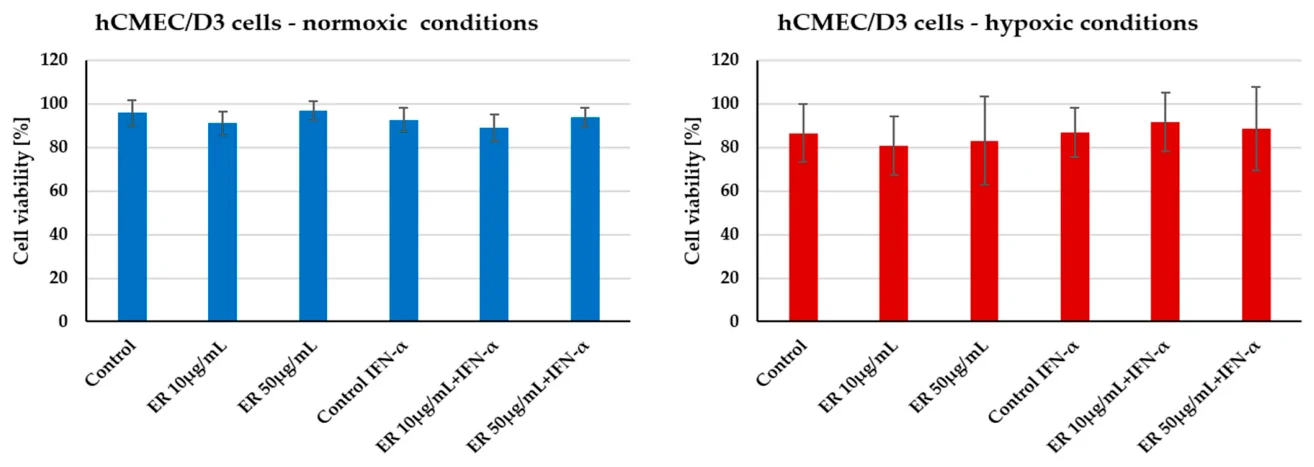
Effects of *R. aculeatus* extract (ER) on the viability of hCMEC/D3 cells determined using the MTT assay. Cells were treated with ER, interferon alpha (IFN-α), or their combination under normoxic (blue) and hypoxic (red) conditions. Data are presented as the mean ± SD. No statistically significant differences were observed (one-way ANOVA followed by Dunnett’s post hoc test, *p* > 0.05).

In the first stage of the study, cell viability was assessed to exclude potential cytotoxic effects of *R. aculeatus* extract and interferon alpha (IFN-α) on hCMEC/D3 cells under normoxic and hypoxic conditions (Figure 2).

Under normoxic conditions, cell viability remained above 89% in all experimental groups. Neither *R. aculeatus* extract, IFN-α, nor their combination significantly reduced cell viability compared with the untreated control. Similarly, under hypoxic conditions, cell viability remained high, ranging from approximately 80% to 91%. This observation is consistent with previous studies demonstrating that moderate hypoxia in hCMEC/D3 cells primarily elicits adaptive responses, resulting in alterations in endothelial phenotype and gene expression rather than overt cytotoxicity [17,18]. No statistically significant differences were detected between the control and treatment groups in normoxia and hypoxia. Overall, the tested concentrations of *R. aculeatus* extract were well tolerated by hCMEC/D3 cells under both normoxic and hypoxic conditions.

#### 2.2.2. Effect of *R. aculeatus* Extract on the Production of Pro-Inflammatory Cytokines

Since chronic venous disorders are associated with persistent endothelial activation and low-grade inflammation, attenuation of inflammatory responses may be beneficial in the management of these conditions. Inflammatory mediators released by activated endothelial cells contribute to leukocyte recruitment, vascular dysfunction, and the progression of venous disease.

**Figure 3:**
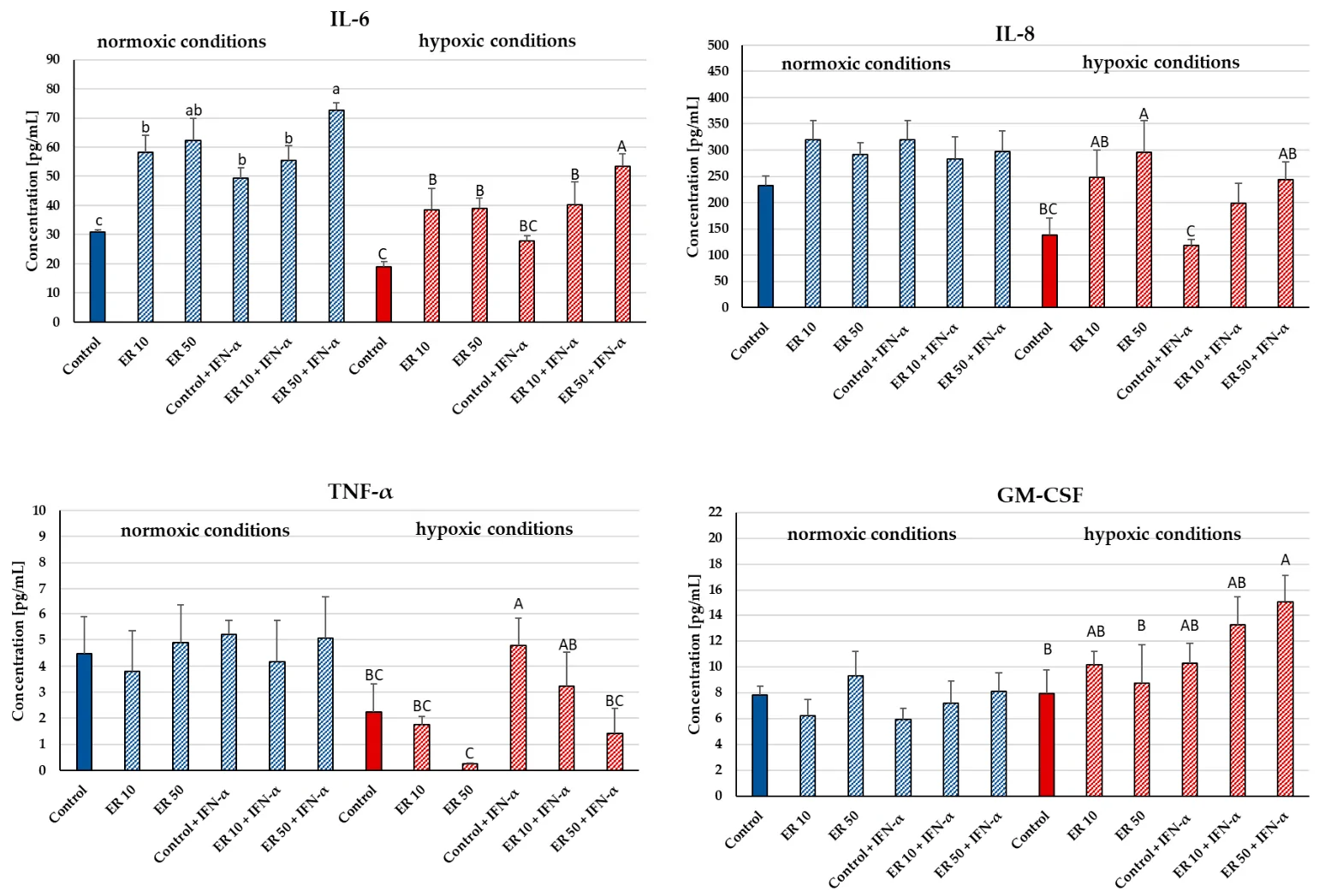
Effect of *Ruscus aculeatus* rhizome extract on cytokine secretion by hCMEC/D3 cells under normoxic and hypoxic conditions. Cells were treated with *R. aculeatus* extract at 10 (ER10) or 50 μg/mL (ER50), alone or in combination with IFN-α (100 U/mL). Data are presented as mean ± SD (*n* = 3). Statistical analysis was performed using two-way ANOVA followed by Tukey’s HSD multiple-comparisons test. Lowercase letters indicate statistical comparisons under normoxic conditions, whereas uppercase letters indicate comparisons under hypoxic conditions. Within each oxygen condition, bars that do not share a common letter are significantly different (*p* < 0.05). Exact adjusted *p*-values are provided in the Supplementary Materials.

To evaluate the effect of *R. aculeatus* extract on the inflammatory response of endothelial cells, the concentrations of selected cytokines involved in endothelial activation and immune regulation were determined. Although the Bio-Plex panel included interleukins: IL-2, IL-4, IL-6, IL-8, IL-10, tumor necrosis factor alpha (TNF-α), interferon gamma (IFN-γ), and granulocyte-macrophage colony-stimulating factor (GM-CSF), only cytokines showing measurable concentrations are presented in the Results section. IL-2, IL-4, IL-10, and IFN-γ were below the limit of detection in all experimental groups. The results are presented in Figure 3.

Under normoxic conditions, treatment with *R. aculeatus* extract, IFN-α alone and in combination with ER resulted in increased secretion of both IL-6 and IL-8 compared with untreated cells. Under hypoxic conditions, the basal concentrations of both cytokines were markedly lower than under normoxia. Treatment with *R. aculeatus* extract increased IL-6 and IL-8 secretion under hypoxic conditions, shifting their concentrations toward those observed under normoxic conditions. GM-CSF secretion was not significantly altered by *R. aculeatus* extract or IFN-α alone under normoxic conditions. Under hypoxic conditions, GM-CSF secretion was significantly higher than in the untreated control only in cells co-treated with 50 μg/mL extract and IFN-α. No significant differences in TNF-α secretion were observed among treatment groups under normoxic conditions.

Under hypoxic conditions, *R. aculeatus* extract alone tended to decrease TNF-α secretion, although the differences compared with the untreated control were not statistically significant. IFN-α significantly increased TNF-α secretion, whereas co-treatment with 50 μg/mL extract significantly attenuated this response.

IL-6, IL-8, TNF-α, and GM-CSF are key mediators involved in endothelial activation, leukocyte recruitment, inflammatory signaling, and communication between endothelial and immune cells. In the present study, *R. aculeatus* extract did not uniformly suppress cytokine secretion, indicating that its effects cannot be described as a broad anti-inflammatory response. This observation is generally consistent with the limited available literature on the anti-inflammatory activity of *R. aculeatus*. For example, Rodrigues et al. reported only moderate inhibition of NO production by *R. aculeatus* preparations in LPS-stimulated RAW264.7 murine macrophages [19]. Interestingly, hypoxia markedly reduced the basal secretion of IL-6 and IL-8 by hCMEC/D3 cells in the present study. This observation is consistent with the context-dependent nature of endothelial responses to hypoxia. Although hypoxia is frequently associated with enhanced inflammatory signaling, its effects on endothelial cytokine production strongly depend on the endothelial cell type, oxygen concentration, and duration of exposure. For example, prolonged hypoxia in HUVECs resulted in an approximately fourfold increase in IL-6 and IL-8 secretion, which was associated with increased endothelial permeability [20]. In contrast, Florczyk et al. demonstrated that hypoxia reduced IL-8 expression in endothelial cells, with HIF-1α exerting an inhibitory effect on IL-8 regulation, whereas HIF-2α produced the opposite response [21]. Moreover, studies performed specifically in hCMEC/D3 cells have shown that exposure to 2% O_2_ for up to 48 h did not significantly alter IL-6 mRNA expression, further indicating that hypoxia does not necessarily enhance inflammatory cytokine responses.

As shown in Figure 3, treatment with *R. aculeatus* extract shifted IL-6 and IL-8 secretion toward the levels observed under normoxic conditions. These findings indicate that the extract modulates endothelial cytokine secretion under hypoxic conditions; however, the functional significance of these changes remains to be established. Unlike IL-6 and IL-8, TNF-α secretion was significantly reduced by *R. aculeatus* extract under hypoxic conditions, particularly at the higher extract concentration. Furthermore, the extract attenuated the increase in TNF-α induced by IFN-α. Since TNF-α is a central mediator of endothelial activation, its reduced secretion suggests that *R. aculeatus* extract can modulate inflammatory responses of endothelial cells under hypoxic conditions.

#### 2.2.3. Effect of *R. aculeatus* Extract on TGF-β Secretion Under Normoxic and Hypoxic Conditions

**Figure 4:**
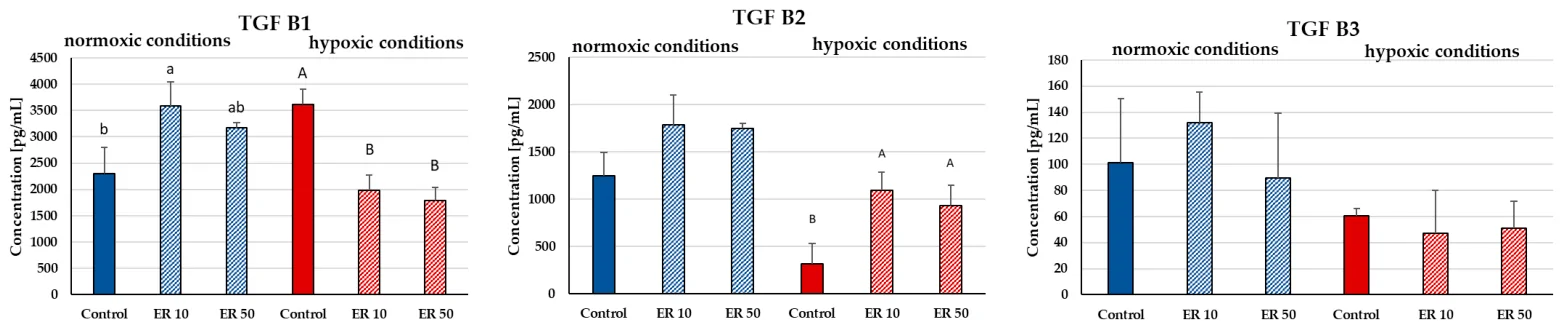
Effect of *Ruscus aculeatus* rhizome extract on TGF secretion by hCMEC/D3 cells under normoxic and hypoxic conditions. Cells were treated with *R. aculeatus* extract at 10 (ER10) or 50 μg/mL (ER50). Data are presented as mean ± SD (*n* = 3). Statistical analysis was performed using two-way ANOVA followed by Tukey’s HSD multiple-comparisons test. Lowercase letters indicate statistical comparisons under normoxic conditions, whereas uppercase letters indicate comparisons under hypoxic conditions. Within each oxygen condition, bars that do not share a common letter are significantly different (*p* < 0.05). Exact adjusted *p*-values are provided in the Supplementary Materials.

Since transforming growth factor beta (TGF-β) isoforms are closely involved in hypoxia-associated endothelial remodeling and vascular adaptation, their secretion was evaluated in relation to oxygen availability and treatment with *R. aculeatus* extract. As shown in Figure 4, the effects of the extract on TGF-β secretion differed among the three isoforms and depended on oxygen conditions.

Exposure of hCMEC/D3 cells to hypoxia resulted in opposite changes in TGF-β1 and TGF-β2 secretion. TGF-β1 levels were significantly increased under hypoxic conditions compared with the normoxic control, whereas TGF-β2 levels were significantly decreased (Table S5). No significant changes in TGF-β3 secretion were observed.

As observed for interleukins, the regulation of individual TGF-β isoforms by hypoxia appears to be highly dependent on the cell and tissue type as well as on the experimental conditions. For example, Saed et al. reported that hypoxia increased TGF-β1 and TGF-β2 mRNA levels in human peritoneal mesothelial cells [22]. Similarly, Akman et al. demonstrated that exposure of human endothelial cells to 1% O_2_ increased TGF-β2 mRNA and protein levels [23]. In contrast, hypoxia decreased TGF-β2 release from retinal Müller cells [24]. Furthermore, Warren et al. observed a marked increase in TGF-β1 expression in osteoblasts exposed to hypoxia, whereas TGF-β2 and TGF-β3 remained unaffected [25]. TGF-β isoforms play important but partly distinct roles in vascular homeostasis. TGF-β1 and TGF-β2 are involved in the regulation of endothelial function, angiogenesis, and vascular remodeling, whereas TGF-β3 is primarily associated with tissue repair and remodeling and is generally considered less profibrotic than TGF-β1.

Under normoxic conditions, treatment with *R. aculeatus* extract increased the secretion of both TGF-β1 and TGF-β2 compared with untreated cells. Interestingly, under hypoxic conditions, treatment with the extract shifted the secretion of both TGF-β1 and TGF-β2 toward the levels observed in normoxic control cells. The extract reduced the hypoxia-associated elevation of TGF-β1 while increasing TGF-β2 from the markedly reduced level observed under hypoxia. This pattern suggests that the extract may counteract hypoxia-associated alterations in TGF-β secretion.

## 3. Materials and Methods

### 3.1. Plant Material and Preparation of the Extract

Rhizomes of *Ruscus aculeatus* were purchased from Flos (Mokrsko, Poland) and ground into a fine powder using a laboratory mill (IKA M20, IKA-Werke GmbH & Co. KG, Staufen, Germany). Extraction was performed by accelerated solvent extraction (ASE) using a Dionex ASE 350 extractor (Thermo Fisher Scientific Inc., Sunnyvale, CA, USA) with an ethanol–water mixture (80:20, *v*/*v*) as the extraction solvent. The extraction temperature was set at 80 °C, with a static extraction time of 10 min. The obtained extract was lyophilized using a Christ Alpha 2–4 LDplus freeze dryer (Martin Christ Gefriertrocknungsanlagen GmbH, Osterode am Harz, Germany) and stored at −20 °C until further analysis.

### 3.2. Phytochemical Characteristics of Extracts

LC-MS analysis was performed using ultra-high performance liquid chromatograph (UHPLC) Infnity Series II with a DAD detector and Agilent 6224 ESI/TOF mass detector (Agilent Technologies, Santa Clara, CA, USA) with the column: Kinetex C18 (Phenomenex, Torrance, CA, USA), 150 mm in length, 2.1 mm inner diameter, and a particle size of 1.7 µm. Chromatographic conditions were as followed: The column thermostat: 30 °C. The flow rate: 0.2 mL/min. The mobile phase consisted of water with 0.05% formic acid (solvent A) and acetonitrile with 0.05% formic acid (solvent B). Gradient elution was performed according to the following program: 0–8 min from 98% A to 93% A, 8–15 min from 93% A to 88%A, 15–29 min from 88% A to 85% A, 29–40 min from 85% A to 80% A, 40–80 min from 80% A to 55% A. LC–MS conditions were as follows: drying gas temperature, 325 °C; drying gas flow rate, 8 L/min; nebulizer pressure, 30 psi; capillary voltage, 3500 V; and skimmer voltage, 65 V. The fragmentor voltage was set at 180 and 280 V in negative-ion mode and at 140 V in positive-ion mode. Compounds were tentatively identified based on accurate mass measurements, characteristic ionization and fragmentation patterns, and comparison with literature data. Molecular formulas were generated from accurate mass data using MassHunter software ver. 10.0 (Agilent Technologies, Santa Clara, CA, USA).

### 3.3. Biological Evaluation

#### 3.3.1. Cell Culture

The human cerebral microvascular endothelial cell line hCMEC/D3 (Cytion GmbH, Heidelberg, Germany) was used as an in vitro model. Cells were cultured in Endothelial Cell Growth Medium (EGM-2, C-22011; Lonza, Basel, Switzerland) supplemented with the EGM-2 BulletKit (CC-3162; Lonza), 2% fetal bovine serum (FBS), 100 U/mL penicillin, and 100 μg/mL streptomycin. Cultures were maintained at 37 °C in a humidified atmosphere containing 5% CO_2_. Cells between passages 5 and 10 were used for all experiments.

#### 3.3.2. Cell Treatment

Cells were seeded into 96-well plates at a density of 2 × 10^4^ cells/well in 200 μL of culture medium and allowed to adhere for 48 h before treatment. Following cell adhesion, hCMEC/D3 cells were treated for 24 h with *Ruscus aculeatus* extract (10 and 50 μg/mL), either alone or in combination with IFN-α (100 U/mL) [26]. Test samples were dissolved in DMSO and further diluted in culture medium to obtain a final DMSO concentration of 0.1%. Cells were incubated under either normoxic conditions (37 °C, 5% CO_2_) or hypoxic conditions (1% O_2_) for 24 h. The hypoxic condition was established based on the experimental protocol described by Patak et al. [17]. All experiments were performed in triplicate, with three biological replicates per experimental condition.

Cell viability was determined using the MTT assay. Following treatment, cells were incubated with MTT solution (5 mg/mL) for 4 h. Formazan crystals were dissolved in DMSO, and absorbance was measured at 550 nm using a microplate reader Eon™ Microplate Spectrophotometer (BioTek, Winooski, VT, USA). Cell viability was expressed as a percentage of the vehicle-treated control (0.1% DMSO).

#### 3.3.3. Cytokine Determination

The concentrations of IL-2, IL-4, IL-6, IL-8, IL-10, TNF-α, IFN-γ, and GM-CSF, as well as TGF-β1, TGF-β2, and TGF-β3, were determined in culture supernatants using Bio-Plex Pro Human Cytokine Group I 8-Plex and Bio-Plex Pro Human TGF-β 3-Plex assays (Bio-Rad Laboratories, Hercules, CA, USA), according to the manufacturer’s instructions. Measurements were performed using a Bio-Plex 3D Suspension Array System and Luminex xPonent Software, Version 4.3 (Bio-Rad), and cytokine concentrations were calculated from standard calibration curves. Samples for TGF-β analysis were acid-activated prior to measurement according to the manufacturer’s protocol. All experiments were performed in triplicate.

### 3.4. Statistical Analysis

Data are presented as mean ± standard deviation (SD) from three independent experiments (*n* = 3). Statistical analyses were performed using two-way analysis of variance (two-way ANOVA), with oxygen condition (normoxia or hypoxia) and treatment as experimental factors, followed by Tukey’s HSD multiple-comparisons test. Differences were considered statistically significant at *p* < 0.05. Exact adjusted *p*-values for multiple comparisons are provided in the Supplementary Materials.

## 4. Conclusions

This study provides new insights into the phytochemical composition of *R. aculeatus* rhizome extract and its effects on human microvascular endothelial cells under normoxic and hypoxic conditions. Phytochemical analysis demonstrated that, in addition to steroidal saponins, which represented the predominant constituents, the ethanolic extract of *R. aculeatus* rhizomes also contained hydroxycinnamic acid amides. The extract differentially modulated endothelial cytokine secretion depending on oxygen availability and the mediator examined. Under hypoxic conditions, it shifted IL-6 and IL-8 secretion toward the levels observed under normoxia and attenuated TNF-α production. The extract also modulated hypoxia-associated alterations in TGF-β secretion, reducing the elevation of TGF-β1 and increasing the markedly decreased TGF-β2 toward normoxic levels. Taken together, these findings suggest that *R. aculeatus* extract acts as a modulator of endothelial secretory responses rather than as a broad suppressor of inflammatory signaling. Such modulation may contribute to the vascular activity attributed to *R. aculeatus*; however, further studies are required to elucidate the underlying molecular mechanisms and determine the relevance of these effects in vascular dysfunction. However, several limitations should be acknowledged. Functional markers of endothelial protection, such as barrier integrity, oxidative stress, or adhesion molecule expression, were not evaluated, and several cytokines included in the multiplex panel remained below the detection limit. In addition, the hypoxic phenotype was not independently confirmed using hypoxia-related molecular markers, such as HIF-1α. Only two extract concentrations and a single 24 h treatment timepoint were evaluated, which precludes conclusions regarding dose–response relationships and the time dependence of the observed effects. Moreover, the biological experiments were performed using the whole R. aculeatus extract; therefore, the observed effects cannot be attributed to any individual phytochemical constituent. Consequently, the observed changes in cytokine secretion should not be interpreted as direct evidence of vasculoprotective activity or as effects of specific extract constituents. Future studies should include a broader range of extract concentrations, multiple treatment timepoints, isolated major constituents, as well as a broader selection of endothelial-relevant mediators. Further studies using peripheral venous endothelial models, particularly primary human venous endothelial cells, are required to confirm the relevance of the observed effects to chronic venous disease.

## Data Availability

Raw, unprocessed data are available from corresponding authors on reasonable request.

## Supplementary Materials

The following supporting information can be downloaded at: https://www.mdpi.com/article/10.3390/molecules31183348/s1, Figure S1. Representative MS and UV–Vis spectra of the major hydroxycinnamic acid amides detected in the *Ruscus aculeatus* rhizome extract; Figure S2. Chromatographic profiles of the *Ruscus aculeatus* rhizome extract obtained by UHPLC–DAD–HRMS: (A) base peak chromatogram (BPC) recorded in negative-ion mode and (B) DAD chromatogram recorded at 205 nm; Figure S3. Representative MS spectrum of Ruscoponticoside E.; Figure S4. UHPLC–HRMS base peak chromatogram (BPC) of the saponin fraction of the *Ruscus aculeatus rhizome* extract recorded in negative-ion mode. Peak numbers correspond to the tentatively identified steroidal saponins listed in Table 2; Table S1. Adjusted *p*-values for pairwise comparisons of IL-6, GM-CSF, TNF-α, and IL-8 secretion under normoxic conditions using Tukey’s HSD multiple-comparisons test. ER10 and ER50—*R. aculeatus* extract at 10 and 50 μg/mL; Table S2. Adjusted *p*-values for pairwise comparisons of IL-6, GM-CSF, TNF-α, and IL-8 secretion under hypoxic conditions using Tukey’s HSD multiple-comparisons test. ER10 and ER50—*R. aculeatus* extract at 10 and 50 μg/mL; Table S3. Adjusted *p*-values for pairwise comparisons of TGF- β1, TGF- β2, and TGF- β3 secretion under normoxic conditions using Tukey’s HSD multiple-comparisons test. ER10 and ER50—*R. aculeatus* extract at 10 and 50 μg/mL; Table S4. Adjusted *p*-values for pairwise comparisons of TGF- β1, TGF- β2, and TGF- β3 secretion under hypoxic conditions using Tukey’s HSD multiple-comparisons test. ER10 and ER50—*R. aculeatus* extract at 10 and 50 μg/mL; Table S5. *p*-values for comparisons of cytokine secretion between normoxic and hypoxic control conditions using Student’s *t*-test.
