# Supplementary material for: Effects of Ruscus aculeatus Rhizome Extract on Human Cerebral Microvascular Endothelial Cells Under Normoxic and Hypoxic Conditions
Source: Molecules. 2026 Sep 21;31(18):3348. doi: 10.3390/molecules31183348 (PMC13609772; doi:10.3390/molecules31183348)
Supplement: Supplementary file 1 [file molecules-31-03348-s001.zip › molecules-4531465-supplementary.pdf]

Effects of *Ruscus aculeatus* Rhizome Extract on Human Cerebral Microvascular Endothelial Cells under Normoxic and Hypoxic Conditions

Magdalena Wójciak[1*], Magdalena Żuk[1], Marcin Feldo[2], Zenon Czuba [3], Anna Kurek-Górecka[3] and Ireneusz Sowa[1*]

[1] Department of Analytical Chemistry, Medical University of Lublin, Chodźki 4a, 20-093 Lublin, Poland; magdalena.wojciak@umlub.edu.pl (M.W.); magdalena.zu25@gmail.com (M.Ż.); ireneusz.sowa@umlub.edu.pl (I.S.).

[2] Chair and Department of Vascular Surgery and Angiology, Medical University of Lublin, 8 Solidarności St., 20-841 Lublin, Poland; martinf@interia.pl

[3] Department of Microbiology and Immunology, Medical University of Silesia in Katowice, Jordana 19, 41-808 Zabrze, Poland; ; zczuba@sum.edu.pl (Z.P.C.); akurekgorecka@sum.edu.pl (A.K-G.);

Correspondence: magdalena.wojciak@umlub.edu.pl) (M.W.); ireneusz.sowa@umlub.edu.pl (I.S.)

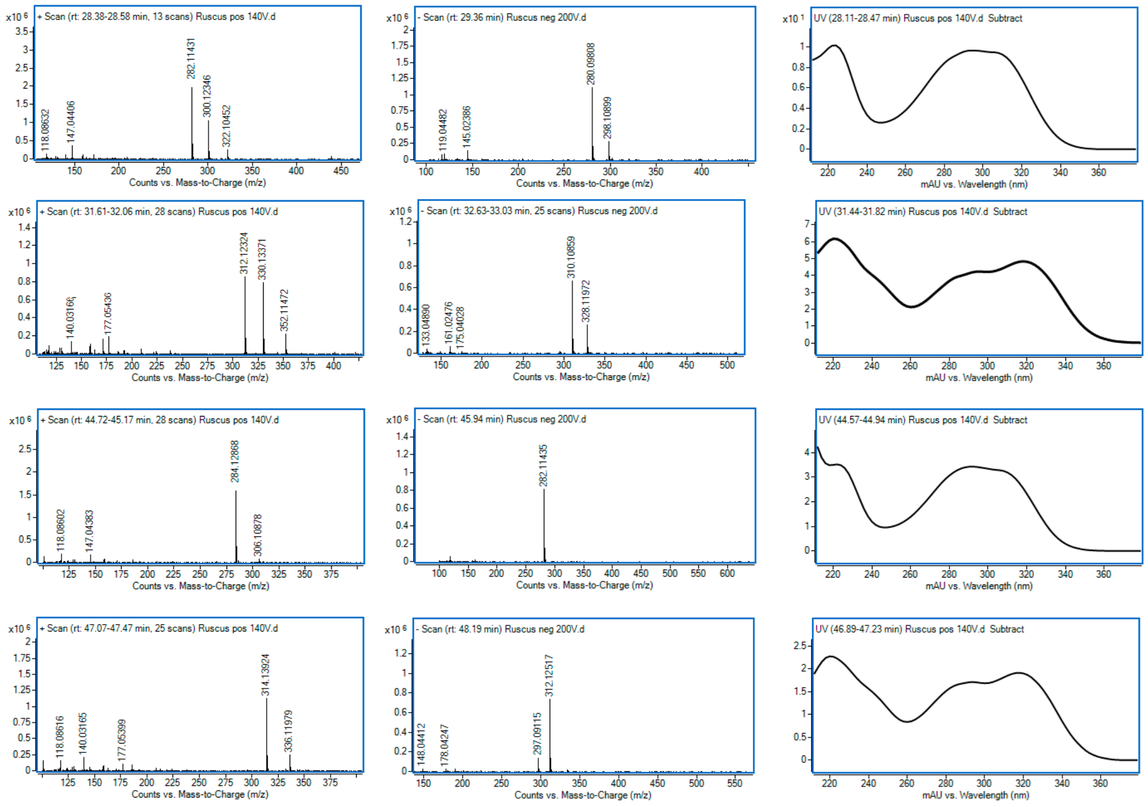

Figure S1. Representative MS and UV–Vis spectra of the major hydroxycinnamic acid amides detected in the *Ruscus aculeatus* rhizome extract.

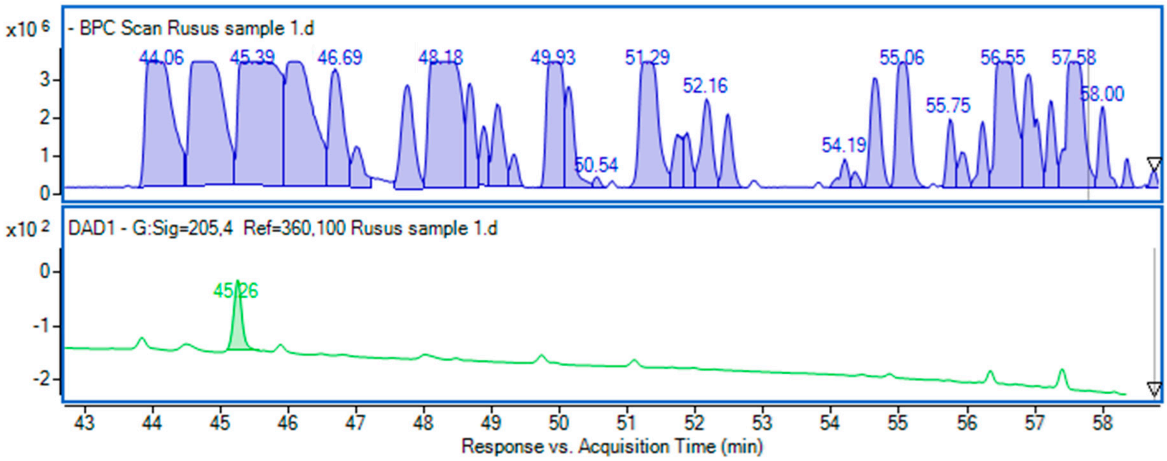

Figure S2. Chromatographic profiles of the *Ruscus aculeatus* rhizome extract obtained by UHPLC–DAD–HRMS: (A) base peak chromatogram (BPC) recorded in negative-ion mode and (B) DAD chromatogram recorded at 205 nm.

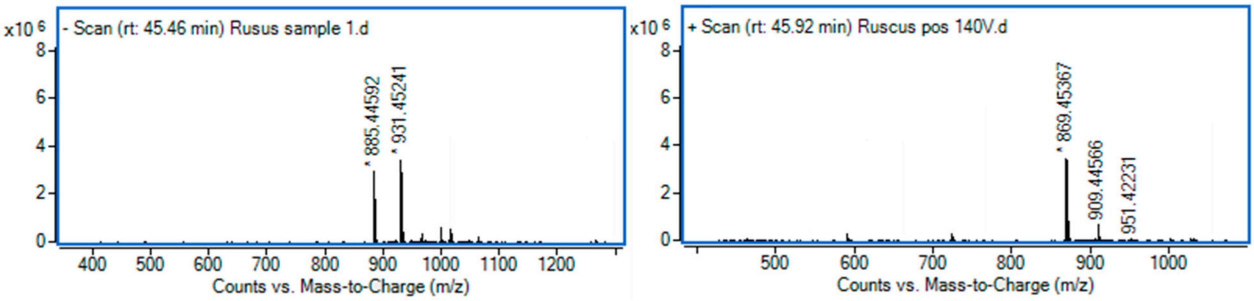

Figure S3. Representative MS spectrum of Ruscoponticoside E.

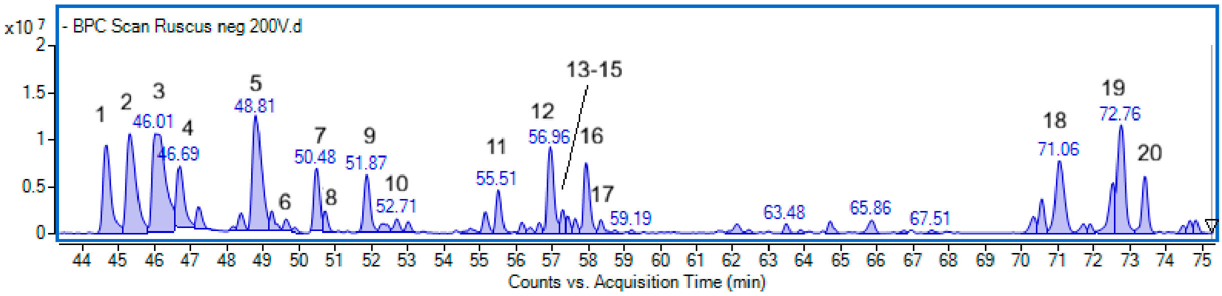

Figure S4. UHPLC–HRMS base peak chromatogram (BPC) of the saponin fraction of the *Ruscus aculeatus* rhizome extract recorded in negative-ion mode. Peak numbers correspond to the tentatively identified steroidal saponins listed in Table 2.

IL-6

	ER10	ER10 + IFN-α	ER50	ER50 + IFN-α	Control	IFN-α
**ER10**		0,976565	0,882471	0,026013	0,000283	0,276645
**ER10 + IFN-α**	0,976565		0,506895	0,007888	0,000537	0,638254
**ER50**	0,882471	0,506895		0,152316	0,00018	0,05128
**ER50 + IFN-α**	0,026013	0,007888	0,152316		0,000159	0,000786
**Control**	0,000283	0,000537	0,00018	0,000159		0,004791
**IFN-α**	0,276645	0,638254	0,05128	0,000786	0,004791	

GM-CSF

	ER10	ER10 + IFN-α	ER50	ER50 + IFN-α	Control	IFN-α
**ER10**		0,950871	0,137035	0,570695	0,731265	0,99976
**ER10 + IFN-α**	0,950871		0,452334	0,957782	0,993289	0,863131
**ER50**	0,137035	0,452334		0,885264	0,753857	0,089006
**ER50 + IFN-α**	0,570695	0,957782	0,885264		0,999696	0,423597
**Control**	0,731265	0,993289	0,753857	0,999696		0,578393
**IFN-α**	0,99976	0,863131	0,089006	0,423597	0,578393	

TNF-α

	ER10	ER10 + IFN-α	ER50	ER50 + IFN-α	Control	IFN-α
**ER10**		0,99958	0,928319	0,869344	0,989949	0,821697
**ER10 + IFN-α**	0,99958		0,9857	0,961136	0,999675	0,935735
**ER50**	0,928319	0,9857		0,999977	0,999141	0,99973
**ER50 + IFN-α**	0,869344	0,961136	0,999977		0,994377	0,999997
**Control**	0,989949	0,999675	0,999141	0,994377		0,986732
**IFN-α**	0,821697	0,935735	0,99973	0,999997	0,986732	

IL-8

	ER10	ER10 + IFN-α	ER50	ER50 + IFN-α	Control	IFN-α
**ER10**		0,766608	0,900774	0,952873	0,062955	1
**ER10 + IFN-α**	0,766608		0,999589	0,996017	0,448536	0,771255
**ER50**	0,900774	0,999589		0,999967	0,306275	0,903883
**ER50 + IFN-α**	0,952873	0,996017	0,999967		0,23775	0,954843
**Control**	0,062955	0,448536	0,306275	0,23775		0,06389
**IFN-α**	1	0,771255	0,903883	0,954843	0,06389	

Table S1. Adjusted p-values for pairwise comparisons of IL-6, GM-CSF, TNF-α, and IL-8 secretion under normoxic conditions using Tukey's HSD multiple-comparisons test. ER10 and ER50 - *R. aculeatus* extract at 10 and 50 μg/mL.

IL-6

	ER10	ER10 + IFN-α	ER50	ER50 + IFN-α	Control	IFN-α
**ER10**		0,998122	0,999999	0,032384	0,004774	0,170018
**ER10 + IFN-α**	0,998122		0,999445	0,064449	0,002512	0,088623
**ER50**	0,999999	0,999445		0,037731	0,004132	0,147761
**ER50 + IFN-α**	0,032384	0,064449	0,037731		0,000172	0,000604
**Control**	0,004774	0,002512	0,004132	0,000172		0,317924
**IFN-α**	0,170018	0,088623	0,147761	0,000604	0,317924	

GM-CSF

	ER10	ER10 + IFN-α	ER50	ER50 + IFN-α	Control	IFN-α
**ER10**	10,17	13,216	8,7467	15,02	7,9133	10,267
**ER10 + IFN-α**		0,482811	0,950151	0,103618	0,749852	1
**ER50**	0,482811		0,149075	0,878156	0,066412	0,514755
**ER50 + IFN-α**	0,950151	0,149075		0,024928	0,995192	0,935422
**Control**	0,103618	0,878156	0,024928		0,010759	0,113757
**IFN-α**	0,749852	0,066412	0,995192	0,010759		0,718222

TNF-α

	ER10	ER10 + IFN-α	ER50	ER50 + IFN-α	Control	IFN-α
**ER10**		0,416521	0,378327	0,996471	0,986365	0,015892
**ER10 + IFN-α**	0,416521		0,017215	0,22109	0,765598	0,356023
**ER50**	0,378327	0,017215		0,632933	0,15429	0,000734
**ER50 + IFN-α**	0,996471	0,22109	0,632933		0,872461	0,007288
**Control**	0,986365	0,765598	0,15429	0,872461		0,04593
**IFN-α**	0,015892	0,356023	0,000734	0,007288	0,04593	

IL-8

	ER10	ER10 + IFN-α	ER50	ER50 + IFN-α	Control	IFN-α
**ER10**		0,698701	0,706581	0,999998	0,061463	0,02205
**ER10 + IFN-α**	0,698701		0,108774	0,753625	0,508284	0,231555
**ER50**	0,706581	0,108774		0,649804	0,005601	0,002163
**ER50 + IFN-α**	0,999998	0,753625	0,649804		0,072554	0,026126
**Control**	0,061463	0,508284	0,005601	0,072554		0,9882
**IFN-α**	0,02205	0,231555	0,002163	0,026126	0,9882	

Table S2. Adjusted p-values for pairwise comparisons of IL-6, GM-CSF, TNF-α, and IL-8 secretion under hypoxic conditions using Tukey's HSD multiple-comparisons test. ER10 and ER50 - *R. aculeatus* extract at 10 and 50 μg/mL.

	TGF- β1			TGF- β2			TGF- β3		
	**Control**	**ER 10**	**ER 50**	**Control**	**ER 10**	**ER 50**	**Control**	**ER 10**	ER 50
**Control**		0,017928	0,081039		0,069774	0,090275		0,666653	0,937632
**ER 10**	0,017928		0,464358	0,069774		0,977453	0,666653		0,479016
**ER 50**	0,081039	0,464358		0,090275	0,977453		0,937632	0,479016	

Table S3. Adjusted p-values for pairwise comparisons of TGF- β1, TGF- β2, and TGF- β3 secretion under normoxic conditions using Tukey's HSD multiple-comparisons test. ER10 and ER50 - *R. aculeatus* extract at 10 and 50 μg/mL.

	TGF- β1			TGF- β2			TGF- β3		
	**Control**	**ER 10**	**ER 50**	**Control**	**ER 10**	**ER 50**	**Control**	**ER 10**	ER 50
**Control**		0,000995	0,000622		0,009167	0,026236		0,754138	0,867932
**ER 10**	0,000995		0,672705	0,009167		0,622498	0,754138		0,973882
**ER 50**	0,000622	0,672705		0,026236	0,622498		0,867932	0,973882	

Table S4. Adjusted p-values for pairwise comparisons of TGF- β1, TGF- β2, and TGF- β3 secretion under hypoxic conditions using Tukey's HSD multiple-comparisons test. ER10 and ER50 - *R. aculeatus* extract at 10 and 50 μg/mL.

Analyte	p value normoxia vs hypoxia
TGF-ß1	0,015918
TGF-ß2	0,007577
TGF-ß3	0,228513
IL-6	0,000412
GM-CSF	0,916045
TNF--α	0,093463
IL-8	0,012423

Table S5. P-values for comparisons of cytokine secretion between normoxic and hypoxic control conditions using Student's t-test.
